# Adaptive School-based Implementation of CBT (ASIC): clustered-SMART for building an optimized adaptive implementation intervention to improve uptake of mental health interventions in schools

**DOI:** 10.1186/s13012-018-0808-8

**Published:** 2018-09-05

**Authors:** Amy M. Kilbourne, Shawna N. Smith, Seo Youn Choi, Elizabeth Koschmann, Celeste Liebrecht, Amy Rusch, James L. Abelson, Daniel Eisenberg, Joseph A. Himle, Kate Fitzgerald, Daniel Almirall

**Affiliations:** 1 Department of Psychiatry, University of Michigan Medical School, Ann Arbor, MI USA; 2 U.S. Department of Veterans Affairs, Quality Enhancement Research Initiative, Washington D.C., USA; 3 School of Public Health, University of Michigan, Ann Arbor, MI USA; 4 School of Social Work, University of Michigan, Ann Arbor, MI USA; 5 Institute for Social Research, University of Michigan, Ann Arbor, MI USA

**Keywords:** Adaptive intervention, Schools, Health behavior change, Cognitive-behavioral therapy

## Abstract

**Background:**

Depressive and anxiety disorders affect 20–30% of school-age youth, most of whom do not receive adequate services, contributing to poor developmental and academic outcomes. Evidence-based practices (EBPs) such as cognitive behavioral therapy (CBT) can improve outcomes, but numerous barriers limit access among affected youth. Many youth try to access mental health services in schools, but school professionals (SPs: counselors, psychologists, social workers) are rarely trained adequately in CBT methods. Further, SPs face organizational barriers to providing CBT, such as lack of administrative support. Three promising implementation strategies to address barriers to school-based CBT delivery include (1) Replicating Effective Programs (REP), which deploys customized CBT packaging, didactic training in CBT, and technical assistance; (2) coaching, which extends training via live supervision to improve SP competence in CBT delivery; and (3) facilitation, which employs an organizational expert who mentors SPs in strategic thinking to promote self-efficacy in garnering administrative support. REP is a relatively low-intensity/low-cost strategy, whereas coaching and facilitation require additional resources. However, not all schools will require all three strategies. The primary aim of this study is to compare the effectiveness of a school-level adaptive implementation intervention involving REP, coaching, and facilitation versus REP alone on the frequency of CBT delivered to students by SPs and student mental health outcomes. Secondary and exploratory aims examine cost-effectiveness, moderators, and mechanisms of implementation strategies.

**Methods:**

Using a clustered, sequential multiple-assignment, randomized trial (SMART) design, ≥ 200 SPs from 100 schools across Michigan will be randomized initially to receive REP vs. REP+coaching. After 8 weeks, schools that do not meet a pre-specified implementation benchmark are re-randomized to continue with the initial strategy or to augment with facilitation.

**Discussion:**

EBPs need to be implemented successfully and efficiently in settings where individuals are most likely to seek care in order to gain large-scale impact on public health. Adaptive implementation interventions hold the promise of providing cost-effective implementation support. This is the first study to test an adaptive implementation of CBT for school-age youth, at a statewide level, delivered by school staff, taking an EBP to large populations with limited mental health care access.

**Trial registration:**

NCT03541317—Registered on 29 May 2018 on ClinicalTrials.gov PRS

**Electronic supplementary material:**

The online version of this article (10.1186/s13012-018-0808-8) contains supplementary material, which is available to authorized users.

## Background

Depression and anxiety disorders are the most common mental health disorders among youth, affecting 20–30% of the population [1]. Evidence-based practices (EBPs), such as cognitive behavioral therapy (CBT), can improve outcomes among youth with these disorders [2–6]. However, less than 20% of youth with depression or anxiety have access to any EBPs, primarily because of limited availability of mental health providers, stigma, and lack of tools to implement effective treatments in the community [7–12]. Even when EBPs such as CBT are offered, fidelity to CBT treatment can be weak [6, 13–16] and most recipients do not receive an adequate therapeutic dose [17]. Without effective treatment, mental health disorders in youth can lead to poor developmental and academic outcomes, substance abuse, self-injury, adult psychopathology, and suicide [2, 18–21], ultimately resulting in immense social and economic costs [2, 18, 22].

EBPs need to be implemented successfully in settings where individuals are most likely to seek care if they are going to have a widespread and meaningful impact on public health. For many individuals with mental health disorders and for youth ages 14 to 21 in particular, non-clinical settings such as schools are attractive options for accessing EBPs [23–25]. Youth primarily spend their time in schools, which typically have school professionals (SPs) with training in social work, counseling, or psychology and who interface with students on a daily basis [22, 26, 27]. Students have reported more willingness to access mental health services at school than in other community settings [10, 11], and among youth who do receive any mental health care, 50–75% receive it exclusively in schools [12, 28]. However, the school professionals with whom they interact rarely have the training or support needed to provide EBPs [29].

Successful implementation of EBPs outside of traditional treatment settings requires scientific determination of optimal implementation strategies that maximize uptake and quality of care by addressing the organizational and community barriers to sustainability. Implementation strategies are highly specified, theory-based methods that target known barriers to improve uptake at provider and system levels [30]. However, implementation strategies designed to improve uptake of CBT among school professionals have not yet been empirically tested on a large scale. SPs do not routinely receive CBT training, and often report low confidence in their ability to deliver such treatments [31–33].

Promising theory-based implementation strategies for improving CBT uptake in schools are Replicating Effective Programs (REP), coaching, and facilitation. These strategies are potentially complementary to each other, but optimal combinations and sequences have not been tested empirically. REP, which is relatively low-burden to end-users [34, 35], focuses on customizing an intervention package to local needs and providing further support through large-group training and ongoing technical assistance [35]. REP has been shown to improve uptake of psychosocial EBPs in community organizations [35–39]; however, may not be sufficient for all providers requiring more supervision in delivering EBPs or for those who are experiencing organizational barriers to EBP adoption [38, 40]. Coaching provides ongoing live supervision of EBP delivery and has shown promise in facilitating CBT adoption in schools [33, 41, 42]. Facilitation includes consultation by an organizational expert in strategic thinking skills for providers to help them enhance organizational and leadership support for CBT implementation at their sites and has been shown to enhance uptake of psychosocial mental health interventions [34, 39].

Currently, there is no research to guide how best to combine REP, coaching, and facilitation for the purpose of CBT implementation in school settings. What is known is that schools, and the SPs that deliver mental health services at the schools, are heterogeneous in terms of barriers to CBT implementation [43]. Optimally efficient CBT uptake in schools may require a “stepped up” type of adaptive implementation intervention, whereby more intensive implementation strategies are only provided to schools that do not respond to a less intensive approach. In addressing barriers to uptake, augmentation of REP with coaching may be essential to overcome SP barriers, while facilitation may help with institutional barriers. Comparative research is needed to best combine these strategies to create and optimize an adaptive implementation intervention that maximizes uptake, cost-effectiveness, and sustainability of an established EBP (CBT), to ultimately improve student mental health.

This study seeks to build the best possible adaptive implementation intervention involving three theory-based implementation strategies—REP, coaching, and facilitation—using a clustered, sequential multiple assignment randomized trial (SMART) design. The study will foster development of an adaptive implementation intervention to improve frequency of CBT delivery to students by SPs, thereby reducing student mental health symptoms [44–49]. The study will take place in high schools across the State of Michigan.

## Methods/Design

### Aims and objectives

#### Primary study aim

The primary aim of this study is to compare the effectiveness of an adaptive implementation intervention on CBT delivery among schools versus REP alone (the control). The adaptive intervention provides schools with REP + coaching from the start and subsequently augments with facilitation for schools needing additional assistance. The primary outcome is the total number of CBT sessions delivered to students by SPs over an 18-month period. Number of CBT sessions is defined further below, and includes group and individual sessions delivered to students.

Specific CBT component delivery and whether delivery of individual or group sessions were brief (< 15 min) or full-length (≥ 15 min) will also be tracked and examined as secondary outcomes. As an exploratory outcome for this primary aim, we will also examine change in student mental health symptoms among students over the study period.

#### Exploratory aims:

1. To estimate the costs of different implementation interventions and determine the incremental cost-effectiveness of added coaching and/or facilitation.
2. To assess whether the effect of augmenting REP with coaching or facilitation is moderated by SP or school factors such as SP knowledge and perceptions of CBT as well as school administrator support of CBT implementation.
3. To determine whether coaching and facilitation improve CBT knowledge, perceptions, skills, or championing skills among SPs, and which of these account for increases in frequency of CBT delivery and improvement in student clinical symptoms.

### Methods

**Fig. 1:**
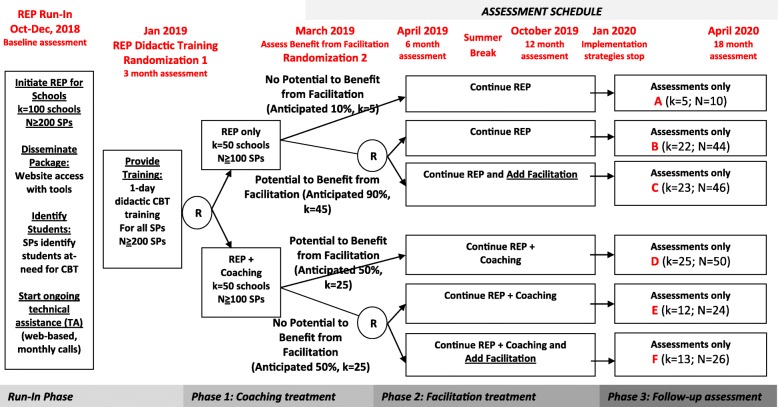
Adaptive implementation of school-based CBT study flow and timeline. Potential to benefit from facilitation is defined as ≥ 1 participating SPs delivering < 3 cognitive behavioral therapy (CBT) components to < 10 students or school professionals (SPs) reporting, on average, > 2 barriers to CBT uptake

This study employs a clustered, sequential multiple assignment randomized trial (SMART) design to inform development of an adaptive implementation intervention (Fig. 1). The study was reviewed and approved by the University of Michigan Institutional Review Board (IRB; UM Protocol # HUM00132239). The study takes advantage of an ongoing initiative to disseminate CBT training in schools in the State of Michigan, the Transforming Research into Action to Improve the Lives of Students (TRAILS) program. All program delivery, training, and implementation support is provided through TRAILS and is considered non-research per local IRBs, and considered exempt from regulation under our approved IRB.

### Setting

The study will take place in high schools across Michigan’s 83 counties, with CBT delivered by existing SPs for students with depression and anxiety. The REP (which includes CBT manual package, training, and technical support for SPs), coaching, and facilitation implementation strategies will be provided through the TRAILS program [31].

### Study design

Figure 1 details the four phases of this study over an 18-month period; the four phases are 3, 2, 10, and 3 months in duration, respectively.

The run-in phase involves deployment of the REP implementation strategy (CBT manual package, training, and technical assistance) and identification of schools, SPs, and potential students in need of CBT by SPs.

For phase 1, eligible schools are randomized with equal probability to continued REP only versus REP combined with coaching (REP+coaching). At the end of phase 1, schools are assessed to determine whether they would potentially benefit from facilitation.

During phase 2, schools that could benefit from facilitation [39] (see Additional file 1: Appendix 1) will be re-randomized in phase 2 with equal probability to continue their implementation strategy from Phase 1 (i.e., REP or REP+coaching) or to have their current strategy augmented with facilitation (i.e., REP+facilitation or REP+coaching+facilitation, respectively). The active elements of the coaching and facilitation strategies will be paused during the summer months (June–August 2019) when schools are not in session.

For phase 3, all implementation strategies will be discontinued. Outcomes will be collected longitudinally throughout all phases, from SPs up to 18 months after the baseline assessment and from students up to 15 months after their baseline assessment.

### Sites/schools

Over 200 SPs from up to 100 schools across the State of Michigan’s 900+ high schools will be recruited by study staff to participate in the study. Every attempt will be made to recruit at least one eligible school from each county in Michigan and to include rural as well as urban and suburban schools.

#### Site inclusion criteria

Schools will be eligible if they

1. Are a high school (grades 9–12) from a school district in one of the 83 counties in Michigan that has not previously participated in a TRAILS CBT training initiative.
2. Are within a 2-h driving distance of a TRAILS coach (who are mental health professionals primarily working in community mental health clinics across Michigan).
3. Agree to participate in data collection throughout the study duration.
4. Identify at least one SP who is eligible and agrees to participate in study assessments throughout the study duration.
5. Allow for SP(s) to deliver individual and/or group mental health support services on school grounds, yet outside of the general education classroom environment.

A school administrator who is a principal or other senior administrator at each participating school will be asked to provide data on building-wide sociodemographics and leadership support for evidence-based practices.

School professionals identified by schools are eligible if they are

1. Employed at a Michigan high school
2. Have a background in clinical school social work, counseling, psychology, or similar field
3. Able to read and understand English and comprehend study assessments

School professionals will be excluded if they have a significant illness or condition that precludes their participation in the implementation strategies, including the REP training and student identification process, coaching, or facilitation, or are unable to provide informed consent for participation in the study activities.

### Student eligibility and recruitment

As part of REP, SPs will be trained during the run-in phase to identify 10 eligible students in need of CBT. Because accurate case finding is critical to successful CBT implementation, training SPs on student identification is a core component of the REP implementation strategy [39] used in previous studies of implementation strategies [40, 50]. SPs will be taught through REP to recognize signs of depression and anxiety in students, using public domain screens (Patient Health Questionnaire 9 modified for teens [PHQ-9T] and generalized anxiety disorder [GAD]-7) [51].

Students are considered eligible if the SP determines they have at least one symptom of depression or anxiety that impacts their daily functioning and well-being. Students are considered ineligible as determined by the SP if they are (1) high school seniors (or would be graduating prior to any CBT sessions); (2) are unable to regularly attend school-delivered CBT skills groups; or (3) are unlikely to benefit from CBT skills groups due to cognitive or developmental disability, lack of English proficiency, or significant behavioral difficulties.

### Stratified randomizations

All randomization occurs at the school level. All study-eligible schools are randomized in phase 1 with equal probability to receive either REP or REP+coaching. In phase 2, schools with documented evidence of a need for additional implementation support based on predetermined criteria (see Additional file 1: Appendix 1) will be further randomized with equal probability to continue their phase 1 strategy (REP or REP+coaching) or to have their current strategy augmented with facilitation (REP+facilitation or REP+coaching+facilitation). To ensure balance across study arms, the first randomization will be stratified based on school size (> 500 or ≤ 500 students), location of school (rural or urban), percentage of students on free/reduced lunch program (≥ %50 or < 50%), and pre-randomization delivery of CBT (any sessions vs. none). The second randomization, among schools that might benefit from facilitation, will be stratified by size, location, and total number of CBT sessions provided in the 8 weeks post first randomization (top 50% vs. bottom 50% within REP or REP+coaching arm).

### Evidence-based practice (EBP) to be implemented

The EBP to be implemented is cognitive behavioral therapy (CBT) for youth with depression or anxiety [52–56]. Modular CBT—defined as individual components of CBT, delivered flexibly and responsively to presenting symptoms [47]—will be utilized in particular, due to its strong evidence base and advantages over other manualized protocols for school-based delivery [45, 49]. Modular CBT has been previously found in several studies to be associated with reduced depressive and anxiety symptoms when compared to usual care [44, 57], and among students in particular [48, 58]. CBT has also been delivered successfully for different racial and ethnic groups [8, 57], thus making it ideal for a statewide trial within schools [59, 60]. Core CBT components used in this study are based on previously established interventions [55, 61] and include psychoeducation, relaxation, instruction in identification and replacement of anxious or depressive thoughts, behavioral activation, creation of fear hierarchies, and exposure. Additional emphasis will be placed on active intervention techniques associated with improved engagement and clinical outcomes, such as agenda setting, modeling of skills, practice with feedback, and assignment of take-home practice activities [62].

### Implementation strategies and components

#### REP

Replicating Effective Programs (REP) [36] will be provided to all schools and is based on Rogers Diffusion Model [63] and social learning theory [64]. REP enhances EBP uptake by customizing interventions to fit the needs of specific settings through EBP packaging (tailoring of the modular CBT manual in user-friendly language), didactic training, and ongoing technical assistance provided by the TRAILS program. The package includes an overview of CBT core components, agendas describing how each component is delivered within a session, sample student screening forms, talking points for students, and suggestions for school-based delivery. REP training to be provided by TRAILS covers modular CBT core components including screening and identification of students. REP technical assistance consists of regular scheduled conference calls during which SPs may receive support from an expert CBT clinician and open access to an interactive website that provides additional resources (e.g., video demonstrations, case simulations) (Table 1).

**Table 1:** Summary of implementation strategies across REP, REP+coaching (REP+C), REP+facilitation (REP+F), and REP+coaching+facilitation (REP+C/F)

Implementation component	REP	REP+C	REP+F	REP+C/F
Replicating Effective Programs (REP)	All sites	All sites randomized	All REP sites that might benefit from facilitation randomized	All REP+C sites that might benefit from facilitation randomized
Step 1: market CBT and disseminate CBT package: a. Recruit schools, disseminate information on CBT program (TRAILS), and R01 study b. Recruit SPs and require they identify 10 students for CBT c. Orient and train SPs to use web tool to track all CBT encounters c. Schedule SP CBT training and program CBT web tool d. Disseminate CBT package (manual + implementation guide) to school professionals (SPs). Implementation guide includes overview of CBT core components (e.g., cognitive restructuring, exposure), session agendas, sample screening forms, talking points, and additional resources.	♦	♦	♦	♦
Step 2: train SPs in CBT 1-day training on the evidence behind CBT and a step-by-step walk-through of core components. Cover common signs of depression and anxiety in students and utilization of public domain screens (e.g., PHQ9T, GAD7).	♦	♦	♦	♦
Step 3: as-needed program assistance and CBT uptake monitoring: bi-weekly conference calls held by REP specialists with an interactive website that provides additional resources (video, case simulations) and Q&A forum led by a REP/CBT expert to address questions regarding clinical content, use of the web tool, manualized materials, and school-based implementation.	♦	♦	♦	♦
Coaching (C)				
CBT expert (coach) attends with SP the CBT sessions delivered to identified students. Coaches will meet with SPs before and/or after each session to address any concerns, questions, or challenges to delivery. a. Weekly pre-session planning by phone or email, direction to appropriate materials and resources, and role-play practice of specific treatment elements b. In vivo modeling of treatment skills during CBT group treatment sessions, observation of SPs’ treatment delivery, post-session discussion of strengths and areas for improvement, and practice of skills with feedback c. Didactic instruction/guided practice of specific skills as needed.		♦		♦
Facilitation (F)				
**S**tep 1: initiation and benchmarking: facilitator with expertise in CBT, implementation methods, education system, and use of EBPs in schools contacts each SP and holds a call with SP to review potential barriers and facilitators to CBT uptake, and set measurable goals for CBT uptake			♦	♦
Step 2: mentoring: facilitator and SP hold regular calls to develop rapport; provides guidance to SP on overcoming specific barriers to CBT uptake by aligning SP strengths with available influence at the school and needs of administrators. If needed, facilitator refers SP to REP TA.			♦	♦
Step 3: leveraging: Facilitator continues calls with SP and with SP reaches out to school administrators, identifies school/community priorities per administration input, and helps SP align CBT use/goals with these existing priorities. Facilitator helps SP summarize and describe added value of CBT to administrators and other school employees (e.g., consistency with other initiatives).			♦	♦
Step 4: ongoing marketing: facilitator, leadership, and SP summarize progress and develop sustainability plans.			♦	♦

*REP* Replicating Effective Programs, *CBT* cognitive behavior therapy, *SP* school professional, *PHQ-9T* Patient Health Questionnaire 9-item Survey for Teens, *GAD-7* Generalized Anxiety Disorder 7-item survey, *EBP* evidence-based practice

♦ represents the presence of the specific implementation component under each implementation strategy to be provided in the study

#### Coaching

The coaching implementation strategy (Table 2) is provided by TRAILS clinicians and is derived from the school-based Positive Behavior Interventions and Supports (PBIS) model of coaching for individual development [65]. Coaching uses a CBT training expert to attend in person to observe group sessions led by the SPs, provide live feedback [66, 67], and model the use of core CBT elements to improve SP competence [65, 68–72]. All SPs from schools randomized to coaching in phase 1 will receive weekly visits from a CBT coach for a minimum of 12 weeks, which will occur in the context of the SPs weekly CBT group. After 12 weeks of on-site coaching, SPs are evaluated on their CBT skill delivery through a short objective competency quiz. SPs deemed to need a full second round of coaching based will receive another full 12 weeks of coaching.

**Table 2:** Fidelity checklist summary for REP, coaching, and facilitation components

Implementation step	Specific implementation tasks	Date completed	Summary of fidelity measure for each component
REP	Step 1: identify schools and SPs, market CBT program		# SP names and contacts, marketing reach (# web hits)
	Step 1: DM disseminates SP and school administrator baseline surveys		# completed surveys
	Step 1: TS orients and trains SPs to use web tool to track all CBT encounters		# SPs at schools receiving package, # website visits
	AN randomizes eligible sites to REP or REP+coaching—phase 1		Complete phase 1 randomization
	Step 2: TS holds training (1 day)		# SPs trained
	Step 2: DM receives list of 10 students/SP		# students listed at each site
	Step 3: TS with TA begin virtual CBT technical assistance phase via regular calls		Call date with SPs, # CBT sessions/site
	Step 3: DM monitors SP uptake of CBT at schools via web tool		# students identified at each school, # CBT sessions
	Step 3: DM starts student assessments		# student assessments completed
	Step 3: TA disseminates school-specific uptake monthly report, eligibility survey		# monthly reports disseminated to each school
	AN determines if site would benefit from facilitation		
	AN randomizes sites that might benefit to add facilitation or not—phase 2		Complete phase 2 randomization for schools that might benefit from facilitation
	Step 3: TA holds as-needed calls with SPs upon request regular conference calls		# conference calls held and attendance
	Step 3: TA sends out regular newsletter highlighting CBT success stories		# newsletters disseminated
Coaching	Coaching fidelity checklist (TRAILS)		
	Step 1: Pre-CBT session to focus on priorities in CBT session		Coach communicates with SP prior to session to identify 2–3 session priorities, provides resources
	Step 2: During CBT session, feedback on session quality		Coach attends student skills group session with SP Role played a skill during session and models skill delivery
			Coach documents if any session components are incomplete/insufficient, and during session models proper delivery during group
			Coach provides 1–2 session strengths and 1–2 session weaknesses to SP, via written or oral feedback
			Coach provides 1–2 suggestions for improvement in delivery, via written or oral feedback, on either CBT skill or overall presentation of group format
	Step 3: Post-CBT-session, preview of upcoming sessions		Coach previews upcoming session goal with SP, provides additional guidance on delivery based on strengths/weaknesses
Facilitation	Step 1 (initiating and benchmarking): facilitator initiates SP calls, identifies barriers, facilitators to CBT implementation		# calls completed with SPs, # minutes/call
	Step 1: facilitator and SP agree on specific uptake goal (e.g., % students completing six sessions)		Facilitator records each site-specific goal, and if met in 6 months
	Step 2 (mentoring): facilitator continues regular calls w/SP, IDs strengths, and influence points; identifies school administrator priorities and additional school champions		Facilitator lists SP’s strengths, linkages to points of influence, school priorities, and champions
	Step 2: facilitator assists SP in aligning strengths/influence with specific CBT uptake goals and advises on aligning strengths to enhance implementation		Facilitator completes action plan linking strengths
	Step 3 (leveraging): facilitator consults with SP’s coach and SP (facilitation + coach arm only) and provides guidance on mitigating barriers to uptake		# consultations with coach
	Step 3: facilitator holds monthly consultation meeting with study staff, coach consultation team		# meetings, minutes created by facilitator
	Step 3: facilitator helps SP summarize and present added value of CBT to administrators		Facilitator records examples of CBT added value
	Step 4 (ongoing marketing): facilitator/SP develop CBT sustainability plan and present to site leadership		Facilitator/SP completes sustainability plan
	Step 4: facilitator refers SPs to additional resources including REP TA		# referrals facilitation made for each site to TA

*TA REP* technical assistant, *TS REP* training specialist, *CC* coach coordinator, *AN* analyst, *DM* database manager, *RA* research assistant

#### Facilitation

Facilitation (Table 3) is based on the Integrated-Promoting Action on Research Implementation in Health Services Framework [73] and promotes provider self-efficacy [74] in mitigating organizational barriers to EBP adoption. Facilitation is delivered via regular phone contact for at least 10 weeks with the SPs by an expert in school and mental health care organization, implementation methods, and use of CBT and EBPs in schools. The facilitator will support SPs in strategic thinking and leadership skills to address organizational barriers covering the following:

1. Initiation and benchmarking (week 1): facilitator contacts each SP to give background on CBT, review potential barriers and facilitators to CBT use (e.g., space to provide CBT, school administration support for the program), and set measurable goals for CBT uptake.
2. Mentoring (weeks 2–9): facilitator and SP hold regular weekly calls to develop rapport; facilitator provides guidance to SP on overcoming specific barriers to CBT uptake by aligning SP strengths with SP available influence at the school and needs of local staff. If needed, facilitator refers SP to REP technical assistant (TA).
3. Leveraging (weeks 2–10): facilitator continues calls with SP and reaches out to school administrators, identifies school/community priorities per administration input, and helps SP align CBT use/goals with these existing priorities. The facilitator helps SP summarize and describe added value of CBT to administrators and other school employees (e.g., consistency with other initiatives).
4. Ongoing marketing (continuous): facilitator, leadership, and SP summarize progress and develop sustainability plans.

**Table 3:** Data sources and measures*

Primary aim:	Measures	Measure frequency	Data sources
Primary outcome and endpoint	Total number of sessions of CBT delivered over the course of 18 months	Weekly, months 1–18 (no collection during summer months)	SP weekly survey
Secondary outcomes	Full sessions of CBT delivered; non-group CBT sessions delivered; brief sessions (< 15 min) of CBT delivered; CBT components delivered	Weekly, months 1–18 (no collection during summer months)	SP weekly survey
Exploratory outcomes	Student mental health outcomes (PHQ-9T; GAD-7)	Months 3, 6, 12, and18	Student survey administered by SP
	Student knowledge of CBT; reported CBT receipt	Months 3, 6, 12, and 18	Student survey administered by SP
Exploratory aim 1: cost effectiveness	Cost of REP, coaching, and facilitation	Weekly, months 1–15; Daily during 2-week time and motion survey	Coach and facilitator logs; REP TA database; SP time and motion survey
	School outcomes (attendance, graduation, GPA); Health services (referrals to care; emergency department admissions)	Student baseline, 6, 12, and 18 months	Student survey administered by SP; academic indicators survey
Exploratory aim 2: moderators	School factors: size, % of students eligible for free/reduced lunch; school administrator support	Baseline	School administrator survey
	SP factors (aggregated): Baseline (run-in): Perceptions of CBT, prior training; time-varying (phase 2): Satisfaction with Phase 1 implementation support, CBT delivery during phase 1, reported barriers to CBT	Baseline, weekly	SP weekly survey; SP survey
Exploratory aim 3: mechanisms	Knowledge, perception, skills, barriers to use; EBPAS, ICS, ILS	Baseline, months 3, 6, 12, and 18	SP survey
	School contextual factors; ILS	Baseline, month 18	School administrator survey
Covariates	Student demographics/behaviors; access to mental health services	Months 3, 6, 12, and 18	Student survey administered by SP
	School factors (attendance, graduation, rates, GPA)	Baseline, month 18	School administrator survey, Academic indicator assessment

*CBT cognitive behavioral therapy, *SP* school professional, *PHQ-9T* Patient Health Questionnaire 9-item Survey for Teens, *GAD-7* Generalized Anxiety Disorder 7-item survey, *REP* Replicating Effective Programs, *EBPAS* Evidence-Based Practice Attitude Scale, *ICS* Implementation Climate Scale, *ILS* Implementation Leadership Scale

### Fidelity monitoring to implementation strategies

Fidelity monitoring will be used to assess whether each site is receiving the core components of each implementation strategy (REP, coaching, and/or facilitation) and to ensure that there is no contamination. Different staff members will serve as REP specialists, coaches, and facilitators. Study staff will train REP specialists, coaches, and facilitators, and meet with them on a regular basis to monitor fidelity. Separate study staff will oversee monitoring of implementation strategy fidelity. Fidelity metrics are described in detail in Table 2. Adequate fidelity to REP is defined by all sites receiving the CBT package, > 90% of SPs receiving training, and at least one monthly contact by the TA specialist to SPs. For coaching, a fidelity checklist [75] will document content covered, post-session feedback provided, session planning and role-play practice that occurred, and provision of resources and materials. The facilitation quantitative fidelity measure [34, 76, 77] will ascertain mode of contact, general content of discussion, and interaction time [39].

### Measures

Data sources and measures (Table 3) will ascertain frequency of CBT session delivery by SPs through month 18 (primary outcome), school-level factors (administrator survey), SP characteristics, and a student outcomes survey. Independent study research associates (RAs) will collect all assessments from SPs and school administrators electronically. To protect student anonymity over the course of the study, SPs themselves will facilitate administration of student surveys, also collected electronically via a secure server that immediately de-identifies all student information.

#### Aim 1 primary outcome (CBT delivery)

The *primary outcome* is the total number of CBT sessions delivered by each SP to students over the course of 18 months. To assess this outcome, SPs will complete a weekly survey where they report their weekly CBT delivery in group or individual sessions, as well as the compnents delivered.  Secondary outcomes will include different types of CBT delivery (individual vs. group; full sessions vs. brief) and delivery of specific CBT components. SPs will be compensated for weekly survey completion in the registry, and study staff will follow up with SPs who do not report CBT delivery for 4 weeks in order to remind them to complete data entry.

#### School-level measures

A longitudinal survey will be given to consenting school administrators to record percentage of students eligible for free/reduced lunch, average classroom size, attendance rate, number of students referred to psychiatric emergency services, and administrator tenure. Administrators will also complete the Implementation Leadership Scale (ILS) [78] to assess institutional support for EBP. No identifying information will be collected as part of these assessments, and no compensation will be provided. Administrators will also be asked to provide approval for participating SPs to collect academic indicator data on GPA, absences, suspensions, and expulsions for participating students.

#### SP characteristics

SPs will also complete longitudinal web-based surveys that include demographic background, level of education, job tenure, prior experience administering CBT, and knowledge and perceptions of CBT delivery using the CBT Knowledge Questionnaire [79]; Provider Attitude Survey [80]; Treatment Manuals Survey [81]; and the Psychotherapy Practice Scale [82]. SPs will be compensated for all completed assessments. SPs will also complete the ILS to ascertain leadership support, and two other validated measures related to support for EBPs—the Implementation Climate Scale (ICS) [83] and the Evidence-Based Practice Attitude Scale (EBPAS) [84].

#### CBT fidelity

Consistent with real-world fidelity monitoring for quality improvement purposes [85], the abovementioned web-based SP weekly assessment will be used to track number of CBT sessions delivered and CBT content delivered each week.

#### Student outcomes

SPs wil be encouraged to identify 10 students that they believe could benefit from CBT prior to and during CBT training. SPs will be trained to create a mini-registry of students using a web-based instrument designed by TRAILS to communicate with other Qualtrics surveys (see Additional file 2: Appendix 2). Students identified by the SP will complete secure electronic surveys on mental health symptoms and health care utilization using the previously described web-based tool (Table 3). SPs will provide to the student in person an information sheet outlining the study eligibility requirements, assessments, compensation, and risks and benefits. A waiver of documentation of consent and waver of parental consent was obtained for ascertaining student outcomes from local IRBs. SPs will be required to provide students with a private location for completing all assessments and will reassure students prior to each assessment that all answers will be de-identified and that they will not have access to the responses. Measures will include student sociodemographic characteristics, health behaviors (e.g., substance use), CBT receipt, knowledge, and use of CBT skills, mental health symptoms (PHQ-9T, GAD-7), and access to mental health services and other healthcare use (e.g., ED referrals or admission). To ensure that students are not coerced into participation, they will be asked to confirm on the web-based survey that they would like to submit their answers. SPs will not be informed if students opt to not submit their answers after completing the survey. Students will be compensated for each survey completed over the 15-month period. In order to protect student privacy from the study staff, SPs will facilitate all student compensation.

#### Cost estimates

For each implementation strategy, we will calculate the average costs and average outcomes per SP using methods described elsewhere [39]. The primary implementation costs are the personnel time spent in REP activities (e.g., SP training, TA), coaching (e.g., time to hire/train coaches, network maintenance, SP coaching time), and facilitation by study participants (including SP and school administrator time). Costs will be quantified as hours multiplied by wages and fringe benefits for each person. Wage rates will be obtained from school records, and in cases where this information is not available, average wages for each occupational level will be used from the Bureau of Labor Statistics. Hours will be tracked through attendance logs for each implementation activity.

To assess costs of delivering CBT, 40 randomly selected SPs will also be asked to complete time-motion surveys for 2 weeks (starting 4 weeks after the phase 2 randomization) that ask about time allotted to providing CBT versus other forms of student counseling, care, or crisis management. School services will be translated to costs based on the wage rates of school providers.

Student-level service costs of CBT delivery and other use will also be estimated from study records of participation in CBT sessions, academic indicators, and self-reported utilization survey data on inpatient, emergency department, and outpatient use outside the school setting. Health care costs will be assigned using Current Procedural Terminology (CPT) codes, and a relative value unit (RVU) weight in the Medicaid Fee Schedule calculates standardized costs in US dollars for each service adjusted for annual levels of inflation using the consumer price index.

### Study sample retention

We will aim to prevent study attrition by following a planned protocol for obtaining the primary research outcome (total CBT sessions delivered by each SP), even if a SP moves to another institution (occurring among < 2% of SPs in our previous studies). A study research assistant (RA) will monitor SP weekly reports of CBT delivery. SPs who fail to submit reports for four consecutive data collection waves will receive two personalized emails from the study RA asking for their report. SPs who do not respond will be contacted by phone by study staff. Study staff will maintain brief communication with all SPs through periods of vacation and will provide easy methods for reporting job transitions that could impact data collection.

### Analyses

All eligible schools, once consented and randomized at phase 1, will be included in an intent-to-treat data analysis sample for all aims. Analyses of student mental health outcomes, however, will be restricted to schools in which at least one SP provided a list of student names for study participation prior to the first randomization. A detailed analysis plan is available in Additional file 3: Appendix 3.

#### Primary aim

The primary aim analysis will determine the effect of the most intensive adaptive implementation intervention, by comparing the total number of CBT sessions delivered by SPs over the course of 18 months between schools receiving REP alone (the control) versus schools receiving the adaptive intervention (REP + coaching + facilitation for schools that are eligible).

#### Exploratory aims

For exploratory aim 1 analyses, incremental cost effectiveness ratios (ICERs) will be calculated for each relevant comparison of implementation interventions by dividing the incremental average costs by the number of CBT sessions delivered as well as the number of depression or anxiety-free days based on PHQ-9T or GAD-7 student score changes between each time point.

Exploratory aim 2 analyses will assess whether the implementation intervention effectiveness is moderated by SP or school-level factors including SP prior training and baseline perceptions of CBT, as well as perceived school administrator support for adoption of CBT. Results of these analyses will be used to construct a more deeply tailored adaptive implementation intervention that further improves uptake, and particularly SP delivery of CBT.

Exploratory aim 3 analyses will test mechanisms through which the coaching and facilitation implementation strategies increase frequency of CBT delivery and/or improve student mental health outcomes.

#### Missing data

Missing outcome data may occur due to school or SP dropout or loss of contact with SPs or students. Our sample retention protocol will ensure that all efforts are made to obtain primary outcome measures for all SPs in all 100 schools. For our primary SP-level outcome, based on preliminary data from TRAILS, we anticipate an attrition rate of < 10%. Prior to conducting all primary and secondary data analyses, missing data will be dealt with explicitly using multiple imputation methods for SMART studies [86, 87].

### Sample size

The estimated sample size for this study is based on our primary aim: a comparison between the expected number of CBT sessions delivered by SPs between months 1 and 18 in schools receiving the adaptive implementation intervention (REP+ coaching + facilitation for schools that are eligible) versus the control (REP only). The sample size calculation for this comparison is a straightforward adjustment to the sample size calculation for a two-sample *t* test [88]. The first adjustment accounts for the clustering of SPs within schools (estimated interclass correlation = 0.03) to account for between-site variation induced by within-site correlation in SP CBT delivery outcomes. The second adjustment accounts for the rate of response following each phase 1 treatment by weighting schools differently to account for some schools being re-randomized and contributing to multiple experimental conditions. Using a two-sided test based on *k* = 100 schools (50 randomized to REP and 50 to REP+coaching in phase 1), on average ≥ 2 SPs per school (anticipated *N* ≥ 200 SPs), a type-1 error rate of 5%, ICC = 0.03, and assuming phase I response rates of 10% (REP only) or 50% (REP+coaching), we will have > 80% power to detect a moderate effect size of *D* = 0.53 between the two implementation interventions on number of CBT sessions delivered.

### Trial status

The study has not started as of August 2018. In the run-in phase (October 2018–January 2019), all eligible SPs will receive Replicating Effective Programs (REP) components, including a 1-day didactic training in CBT in mid-January of 2019. The first randomization will occur in late January 2019.

## Discussion

To our knowledge, this is the first study to comparatively test adaptive implementation interventions at a population (state) level to promote utilization of a modular CBT intervention outside of traditional clinical settings, as delivered by existing school staff, for school-age youth with depressive and/or anxiety symptoms. This is also the first type III hybrid implementation-effectiveness trial to use a SMART design that seeks to understand how best to sequence three implementation strategies (REP, coaching, and facilitation) to improve SP-delivered CBT and student mental health outcomes. The study also informs the more efficient use of implementation resources as not all schools may require the most intensive implementation strategy. In certain contexts, REP alone may significantly improve uptake of evidence-based preventive health interventions, particularly when financial incentives also support their use. However, in other contexts, optimal uptake will require an approach that augments REP with a more intensive implementation strategy such as facilitation. This SMART design will determine the best way to tailor delivery of more intensive implementation strategies (e.g., coaching, facilitation) to schools that need more than initial REP, and can also yield a more cost-effective approach.

This study also incorporates implementation strategies from differing theoretical foundations to better understand links between the various strategies and different mechanisms which can be targeted to overcome barriers to EBP uptake, hence, ultimately leading to more precise implementation. Notably, combining REP, facilitation, and coaching to optimize CBT implementation in school settings provides an innovative way to address provider and organizational barriers, potentially maximizing EBP uptake and impact on student outcomes. In addition to determining optimal implementation strategies to embed CBT in schools for youth with depression or anxiety, this work will elucidate mechanisms of successful implementation to inform the customization of these strategies based on factors specific to different providers, organizations, and communities. Coaching and facilitation both have proven valuable and target different barriers, but the mechanisms by which they foster EBP uptake remain unknown. This study will also help elucidate if and how these implementation strategies foster frontline provider leadership, including transformational and transactional leadership skills previously studied in health care settings. Cost-effectiveness analyses will further tie differences in the cost of an implementation strategy (or adaptive sequence of strategies) to differences in important student behavioral and academic outcomes, including other forms of health care utilization and high school graduation rates.

Results from this study will also provide insight into whether improved CBT knowledge, perceptions, or skills among SPs are associated with increases in fidelity to CBT delivery and improved student outcomes. Understanding the mechanisms by which specific implementation strategies such as facilitation and coaching impact EBP uptake will inform their more precise use in different settings. The sequential randomizations embedded in the SMART design allow us to consider how different school and SP-level factors change over the course of the study and moderate the effectiveness of implementation strategies. These moderation effects can more specifically inform tailored and targeted implementation strategies improving provision of implementation support to schools as their needs change over time, and informing construction of the *most effective adaptive implementation intervention* for improving mental health outcomes across states and school districts.

Despite the novel design of this study, as well as the comprehensive assessment of implementation strategies, there are limitations that warrant consideration. Notably, the opportunity to use a state-wide network of SPs and coaches to implement CBT precluded in-person data collection from students, which would have led to unsustainable study costs. We are also limited to enrolling schools based on availability of TRAILS-trained coaches. We considered several alternative designs that could be applied to large-scale implementation of school-based CBT. The SMART design used here, however, allows us to make this comparison *as well as* understand whether and how coaching and facilitation work with each other to impact implementation outcomes. Further, the sequential randomizations included in the SMART design allow testing of potential time-varying moderators or how effectiveness of phase 2 implementation strategies differs by change in key metrics during phase 1. Understanding these dynamics enriches our understanding as to which schools benefit most from different implementation strategies and also informs potential mechanisms for change under different implementation strategies.

## Conclusions

Overall, the proposed study addresses two major public health priorities in mental health services implementation: (1) reducing the provider capacity shortage affecting school-age youth (i.e., increasing the number of trained mental health providers that reach youth in underserved regions by increasing access to quality mental health therapies by utilizing school settings); and (2) enhancing the scientific knowledge base of implementation science by determining the optimal adaptive strategies for promoting the uptake of EBPs in community-based settings. This study will also support the deployment of a sustainable infrastructure capable of disseminating evidence-based mental health practices across an entire state’s public school system and determine an optimal, adaptive strategy for cost-effective utilization of this implementation infrastructure. Ultimately, the sustainability of the study is potentially realized through a state-wide system that can effectively train existing SPs in EBPs, with capacity to continuously and rapidly update SPs as advancing clinical science provides improved treatments. This work has the potential to give hard-to-reach students rapid access to the latest treatments and treatment advances, by creating an adaptive implementation intervention that can potentially be scaled up and spread nationally.

## Additional files

Additional file 1: Appendix 1 Determining need for facilitation (randomization criteria). (DOCX 13 kb)

Additional file 2: Appendix 2 School Professional Outcomes Tool. (DOCX 20 kb)

Additional file 3: Appendix 3 Analysis plan. (DOCX 12 kb)

## Abbreviations

ADEPT: Adaptive Implementation of Effective Programs Trial
ASIC: Adaptive School-based Implementation of CBT
CBT: Cognitive behavioral therapy
CPT: Current procedural terminology
EBP: Evidence-based practices
EBPAS: Evidence-Based Practice Attitude Scale
GAD-7: General Anxiety Disorder 7-item
GPA: Grade point average
ICS: Implementation Climate Scale
ILS: Implementation Leadership Scale
PBIS: Positive Behavior Interventions and Supports
PHQ-9T: Patient Health Questionnaire 9 modified for teens
RA: Research associate
REP: Replicating Effective Programs
ROC: Receiver-operating curve
RVU: Relative value unit
SMART: Sequential multiple-assignment randomized trial
SP: School professional (counselors, psychologists, social workers)
TA: Technical assistant
TRAILS: Transforming Research into Action to Improve the Lives of Students

## References

[1] Merikangas KR, He JP, Burstein M, Swanson SA, Avenevoli S, Cui LH, Benjet C, Georgiades K, Swendsen J (2010). Lifetime prevalence of mental disorders in U.S. adolescents: results from the National Comorbidity Survey Replication-Adolescent Supplement (NCS-A). J Am Acad Child Psy.

[2] Charvat JL (2012). Research on the relationship between mental health and academic achievement. National Association of School Pscyhologists.

[3] Greenberg PE, Kessler RC, Birnbaum HG, Leong SA, Lowe SW, Berglund PA, Corely-Lisle PL (2003). The economic burden of depression in the United States: how did it change between 1990 and 2000?. J Clin Psychiatry.

[4] Smyth JM, Arigo D (2009). Recent evidence supports emotion-regulation interventions for improving health in at-risk and clinical populations. Current opinion in psychiatry.

[5] Weisz JR, Southam-Gerow MA, Gordis EB, Connor-Smith JK, Chu BC, Langer DA, McLeod BD, Jensen-Doss A, Updegraff A, Weiss B (2009). Cognitive-behavioral therapy versus usual clinical care for youth depression: an initial test of transportability to community clinics and clinicians. J Consult Clin Psychol.

[6] Zins JE, Bloodworth MR, Weissberg RP, Walberg HJ, Zins JE, Weissberg RP, Wang MC, Walberg HJ (2004). The scientific base linking emotional learning to student success and academic outcomes. Building academic success on social and emotional learning: What does the research say?.

[7] Ginsburg GS, Becker KD, Drazdowski TK, Tein JY (2012). Treating anxiety disorders in inner city schools: results from a pilot randomized controlled trial comparing CBT and usual care. Child Youth Care Forum.

[8] Huey SJ, Polo AJ (2008). Evidence-based psychosocial treatments for ethnic minority youth. J Clin Child Adolesc Psychol.

[9] Kataoka SH, Zhang L, Wells KB (2002). Unmet need for mental health care among U.S. children: variation by ethnicity and insurance status. Am J Psychiatry.

[10] Farmer EM, Burns BJ, Phillips SD, Angold A, Costello EJ (2003). Pathways into and through mental health services for children and adolescents. Psychiatr Serv.

[11] Burns BJ, Costello EJ, Angold A, Tweed D, Stangl D, Farmer E, Erkanli A (1995). Children's mental health service use across service sectors. Health Aff.

[12] Weist MD, Rubin M, Moore E, Adelsheim S, Wrobel G (2007). Mental health screening in schools. J Sch Health.

[13] Hallfors D, Godette D (2002). Will the ‘principles of effectiveness’ improve prevention practice? Early findings from a diffusion study. Health Educ Res.

[14] Forman SG, Olin SS, Hoagwood KE, Crowe M, Saka N (2008). Evidence-based interventions in schools: developers’ views of implementation barriers and facilitators. Sch Ment Heal.

[15] Rones M, Hoagwood K (2000). School-based mental health services: a research review. Clin Child Fam Psychol Rev.

[16] Atkins MS, Frazier SL, Abdul-Adil J, Talbott E, Weist MD, Evans SW, Lever NA (2003). School-based mental health services in urban communities. Handbook of school mental health, advancing practice and research.

[17] Garland AF, Brookman-Frazee L, Hurlburt MS, Accurso EC, Zoffness RJ, Haine-Schlagel R, Ganger W (2010). Mental health care for children with disruptive behavior problems: a view inside therapists’ offices. Psychiatr Serv.

[18] Asarnow JR, Jaycox LH, Duan N, LaBorde AP, Rea MM, Tang L, Anderson M, Murray P, Landon C, Tang B (2005). Depression and role impairment among adolescents in primary care clinics. J Adolesc Health.

[19] Jaycox LH, Langley AK, Stein BD, Wong M, Sharma P, Scott M, Schonlau M (2009). Support for students exposed to trauma: a pilot study. Sch Ment Heal.

[20] Edmunds JM, Beidas RS, Kendall PC. Dissemination and implementation of evidence-based practices: training and consultation as implementation strategies. Clin Psychol. 2013;20(2):152–65. 10.1111/cpsp.12031 PMC3780425 24072959

[21] Mychailyszyn MP, Bedas RS, Benjamin CL, Edmunds JM, Podell JL, Cohen JS, Kendall PC (2011). Assessing and treating child anxiety in schools. Psychol Sch.

[22] Bruns EJ, Duong MT, Lyon AR, Pullmann MD, Cook CR, Cheney D, McCauley E (2016). Fostering SMART partnerships to develop an effective continuum of behavioral health services and supports in schools. Am J Orthopsychiatry.

[23] Garrison EG, Roy IS, Azar V (1999). Responding to the mental health needs of Latino children and families through school-based services. Clin Psychol Rev.

[24] Langley AK, Nadeem E, Kataoka SH, Stein BD, Jaycox LH (2010). Evidence-based mental health programs in schools: barriers and facilitators of successful implementation. Sch Ment Heal.

[25] Merry S, McDowell H, Wild CJ, Bir J, Cunliffe R (2004). A randomized placebo-controlled trial of a school-based depression prevention program. J Am Acad Child Adolesc Psychiatry.

[26] Stephan SH, Weist M, Kataoka S, Adelsheim S, Mills C (2007). Transformation of children’s mental health services: the role of school mental health. Psychiatr Serv.

[27] Schwebel DC, Plumert JM, Pick HL (2000). Integrating basic and applied developmental research: a new model for the twenty-first century. Child Dev.

[28] Green JG, McLaughlin KA, Alegria M, Costello EJ, Gruber MJ, Hoagwood K, Leaf PJ, Olin S, Sampson NA, Kessler RC (2013). School mental health resources and adolescent mental health service use. J Am Acad Child Adolesc Psychiatry.

[29] Beidas RS, Barmish AJ, Kendall PC (2009). Training as usual: can therapist behavior change after reading a manual and attending a brief workshop on cognitive behavioral therapy for youth anxiety?. Behav Ther.

[30] Powell BJ, Waltz TJ, Chinman MJ, Damschroder LJ, Smith JL, Matthieu MM, Proctor EK, Kirchner JE (2015). A refined compilation of implementation strategies: results from the Expert Recommendations for Implementing Change (ERIC) project. Implement Sci.

[31] Koschmann EA,J, Fitzgerald K, Kilbourne AM (2017). Transforming research into action to improve the lives of students (TRAILS).

[32] Nadeem E, Gleacher A, Beidas RS (2013). Consultation as an implementation strategy for evidence-based practices across multiple contexts: unpacking the black box. Adm Policy Ment Hlth.

[33] Beidas RS, Mychailyszyn MP, Edmunds JM, Khanna MS, Downey MM, Kendall PC (2012). Training school mental health providers to deliver cognitive-behavioral therapy. Sch Ment Heal.

[34] Kilbourne AM, Goodrich DE, Lai Z, Almirall D, Nord KM, Bowersox NW, Abraham KM (2015). Reengaging veterans with serious mental illness into care: preliminary results from a national randomized trial. Psychiatr Serv.

[35] Kelly JA, Heckman TG, Stevenson LY, Williams PN, Ertl T, Hays RB, Leonard NR, O'Donnell L, Terry MA, Sogolow ED (2000). Transfer of research-based HIV prevention interventions to community service providers: fidelity and adaptation. AIDS Educ Prev.

[36] Kilbourne AM, Neumann MS, Pincus HA, Bauer MS, Stall R (2007). Implementing evidence-based interventions in health care: application of the replicating effective programs framework. Implement Sci.

[37] Kilbourne AM, Neumann MS, Waxmonsky J, Bauer MS, Kim HM, Pincus HA, Thomas M (2012). Public-academic partnerships: evidence-based implementation: the role of sustained community-based practice and research partnerships. Psychiatr Serv.

[38] Waxmonsky J, Kilbourne AM, Goodrich DE, Nord KM, Lai Z, Laird C, Clogston J, Kim HM, Miller C, Bauer MS (2014). Enhanced fidelity to treatment for bipolar disorder: results from a randomized controlled implementation trial. Psychiatr Serv.

[39] Kilbourne AM, Almirall D, Eisenberg D, Waxmonsky J, Goodrich DE, Fortney JC, Kirchner JE, Solberg LI, Main D, Bauer MS (2014). Protocol: adaptive implementation of effective programs trial (ADEPT): cluster randomized SMART trial comparing a standard versus enhanced implementation strategy to improve outcomes of a mood disorders program. Implement Sci.

[40] Kilbourne AM, Almirall D, Goodrich DE, Lai Z, Abraham KM, Nord KM, Bowersox NW (2014). Enhancing outreach for persons with serious mental illness: 12-month results from a cluster randomized trial of an adaptive implementation strategy. Implement Sci.

[41] Owens JS, Lyon AR, Brandt NE, Warner CM, Nadeem E, Spiel C, Wagner M (2014). Implementation science in school mental health: key constructs in a developing research agenda. Sch Ment Heal.

[42] Eiraldi R, Wolk CB, Locke J, Beidas R (2015). Clearing hurdles: the challenges of implementation of mental health evidence-based practices in under-resourced schools. Adv School Ment Health Promot.

[43] Beidas RS, Kendall PC (2010). Training therapists in evidence-based practice: a critical review of studies from a systems-contextual perspective. Clin Psychol-Sci Pr.

[44] Lyon AR, Charlesworth-Attie S, Vander Stoep A, McCauley E (2011). Modular psychotherapy for youth with internalizing problems: implementation with therapists in school-based health centers. Sch Psychol Rev.

[45] Becker EM, Becker KD, Ginsburg GS (2012). Modular cognitive behavioral therapy for youth with anxiety disorders: a closer look at the use of specific modules and their relation to treatment process and response. Sch Ment Heal.

[46] Chiu AW, Langer DA, McLeod BD, Har K, Drahota A, Galla BM, Jacobs J, Ifekwunigwe M, Wood JJ (2013). Effectiveness of modular CBT for child anxiety in elementary schools. Sch Psychol Q.

[47] Chorpita BF, Weisz JR, Daleiden EL, Schoenwald SK, Palinkas LA, Miranda J, Higa-McMillan CK, Nakamura BJ, Austin AA, Borntrager CF (2013). Long-term outcomes for the child STEPs randomized effectiveness trial: a comparison of modular and standard treatment designs with usual care. J Consult Clin Psychol.

[48] Chorpita BF, Daleiden EL, Park AL, Ward AM, Levy MC, Cromley T, Chiu AW, Letamendi AM, Tsai KH, Krull JL: Child STEPs in California: a cluster randomized effectiveness trial comparing modular treatment with community implemented treatment for youth with anxiety, depression, conduct problems, or traumatic stress. 2016. 10.1037/ccp0000133 27548030

[49] Chiu AW, Langer DA, McLeod BD, Har K, Drahota A, Galla BM, Jacobs J, Ifekwunigwe M, Wood JJ (2013). Effectiveness of modular CBT for child anxiety in elementary schools. Sch Psychol.

[50] Kilbourne AM, Almirall D, Eisenberg D, Waxmonsky J, Goodrich DE, Fortney JC, Kirchner JE, Solberg LI, Main D, Bauer MS (2014). Protocol: adaptive implementation of effective programs trial (ADEPT): cluster randomized SMART trial comparing a standard versus enhanced implementation strategy to improve outcomes of a mood disorders program. Implement Sci.

[51] Richardson LP, McCauley E, Grossman DC, McCarty CA, Richards J, Russo JE, Rockhill C, Katon W (2010). Evaluation of the Patient Health Questionnaire-9 item for detecting major depression among adolescents. Pediatrics.

[52] March J, Silva S, Petrycki S, Curry J, Wells K, Fairbank J, Burns B, Domino M, McNulty S, Vitiello B (2004). Fluoxetine, cognitive-behavioral therapy, and their combination for adolescents with depression: Treatment for Adolescents with Depression Study (TADS) randomized controlled trial. JAMA.

[53] Walkup JT, Albano AM, Piacentini J, Birmaher B, Compton SN, Sherrill JT, Ginsburg GS, Rynn MA, McCracken J, Waslick B (2008). Cognitive behavioral therapy, sertraline, or a combination in childhood anxiety. N Engl J Med.

[54] Hofmann SG, Asnaani A, Vonk IJ, Sawyer AT, Fang A (2012). The efficacy of cognitive behavioral therapy: a review of meta-analyses. Cogn Ther Res.

[55] Chorpita BF, Daleiden EL, Weisz JR (2005). Modularity in the design and application of therapeutic interventions. Appl Prev Psychol.

[56] Chorpita BF WJ. Modular approach to therapy for children with anxiety, depression, or conduct problems. Cambridge: University of Hawaii at Manoa and Judge Baker Children's Center, Harvard medical school; 2005.

[57] Ginsburg GS, Becker KD, Kingery JN, Nichols T (2008). Transporting CBT for childhood anxiety disorders into inner-city school-based mental health clinics. Cogn Behav Pract.

[58] Weisz JR, Chorpita BF, Palinkas LA, Schoenwald SK, Miranda J, Bearman SK, Daleiden EL, Ugueto AM, Ho A, Martin J (2012). Testing standard and modular designs for psychotherapy treating depression, anxiety, and conduct problems in youth: a randomized effectiveness trial. Arch Gen Psychiatry.

[59] Masia-Warner C, Klein RG, Dent HC, Fisher PH, Alvir J, Albano AM, Guardino M (2005). School-based intervention for adolescents with social anxiety disorder: results of a controlled study. J Abnorm Child Psychol.

[60] Herzig-Anderson K, Colognori D, Fox JK, Stewart CE, Masia Warner C (2012). School-based anxiety treatments for children and adolescents. Child Adolesc Psychiatr Clin N Am.

[61] Chorpita BF, Daleiden EL (2009). Mapping evidence-based treatments for children and adolescents: application of the distillation and matching model to 615 treatments from 322 randomized trials. J Consult Clin Psychol.

[62] Garland AF, Hawley KM, Brookman-Frazee L, Hurlburt MS (2008). Identifying common elements of evidence-based psychosocial treatments for children's disruptive behavior problems. J Am Acad Child Adolesc Psychiatry.

[63] Rogers EM (2003). Diffusion of innovations, 5th edn.

[64] Green LW, Kreuter MW. Health promotion planning: an educational and environmental approach. In: Health promotion planning: an educational and environmental approach. Edn. California City: Mayfield Publishing Company; 1991.

[65] Hershfeldt PA, Pell K, Sechrest R, Pas ET, Bradshaw CP (2012). Lessons learned coaching teachers in behavior management: the PBISplus coaching model. J Educ Psychol Consult.

[66] Fixsen DL, Blase KA, Duda MA, Naoom SF, Van Dyke M (2010). Implementation of evidence-based treatments for children and adolescents: research findings and their implications for the future.

[67] Herschell AD, Kolko DJ, Baumann BL, Davis AC (2010). The role of therapist training in the implementation of psychosocial treatments: a review and critique with recommendations. Clin Psychol Rev.

[68] McHugh RK, Barlow DH (2010). The dissemination and implementation of evidence-based psychological treatments. A review of current efforts. Am Psychol.

[69] Lochman JE, Boxmeyer C, Powell N, Qu L, Wells K, Windle M (2009). Dissemination of the coping power program: importance of intensity of counselor training. J Consult Clin Psychol.

[70] Miller WR, Yahne CE, Moyers TB, Martinez J, Pirritano M (2004). A randomized trial of methods to help clinicians learn motivational interviewing. J Consult Clin Psychol.

[71] Joyce BR, Showers B: Student achievement through staff development, 3rd edn. Alexandria, VA: Association for Supervision and Curriculum Development; 2002.

[72] Funderburk B, Chaffin M, Bard E, Shanley J, Bard D, Berliner L (2015). Comparing client outcomes for two evidence-based treatment consultation strategies. J Clin Child Adolesc Psychol.

[73] Kilbourne AM, Abraham KM, Goodrich DE, Bowersox NW, Almirall D, Lai Z, Nord KM (2013). Cluster randomized adaptive implementation trial comparing a standard versus enhanced implementation intervention to improve uptake of an effective re-engagement program for patients with serious mental illness. Implement Sci.

[74] Bandura A (1977). Self-efficacy: toward a unifying theory of behavioral change. Psychol Rev.

[75] Marchese DD, Becker KD, Keperling JP, Domitrovich CE, Reinke WM, Embry DD, Ialongo NS: A Step-by-step guide for coaching classroom teachers in evidence-based interventions: Oxford University Press; 2017.

[76] Kilbourne AM, Abraham KM, Goodrich DE, Bowersox NW, Almirall D, Lai Z, Nord KM (2013). Cluster randomized adaptive implementation trial comparing a standard versus enhanced implementation intervention to improve uptake of an effective re-engagement program for patients with serious mental illness. Implement Sci.

[77] Kirchner JE, Ritchie MJ, Pitcock JA, Parker LE, Curran GM, Fortney JC (2014). Outcomes of a partnered facilitation strategy to implement primary care–mental health. J Gen Intern Med.

[78] Aarons GA, Ehrhart MG, Farahnak LR (2014). The Implementation Leadership Scale (ILS): development of a brief measure of unit level implementation leadership. Implement Sci.

[79] Kolko DJ, Baumann BL, Herschell AD, Hart JA, Holden EA, Wisniewski SR (2012). Implementation of AF-CBT by community practitioners serving child welfare and mental health: a randomized trial. Child Maltreat.

[80] Kramer TL, Burns BJ (2008). Implementing cognitive behavioral therapy in the real world: a case study of two mental health centers. Implement Sci.

[81] Addis ME, Krasnow AD (2000). A national survey of practicing psychologists’ attitudes toward psychotherapy treatment manuals. J Consult Clin Psychol.

[82] Hepner KA, Azocar F, Greenwood GL, Miranda J, Burnam MA (2010). Development of a clinician report measure to assess psychotherapy for depression in usual care settings. Admin Pol Ment Health.

[83] Ehrhart MG, Aarons GA, Farahnak LR (2014). Assessing the organizational context for EBP implementation: the development and validity testing of the Implementation Climate Scale (ICS). Implement Sci.

[84] Aarons GA (2004). Mental health provider attitudes toward adoption of evidence-based practice: the Evidence-Based Practice Attitude Scale (EBPAS). Ment Health Serv Res.

[85] Bauer MS, McBride L, Williford WO, Glick H, Kinosian B, Altshuler L, Beresford T, Kilbourne AM, Sajatovic M, Cooperative Studies Program 430 Study T (2006). Collaborative care for bipolar disorder: part II. Impact on clinical outcome, function, and costs. Psychiatr Serv.

[86] Shortreed SM, Laber E, Scott Stroup T, Pineau J, Murphy SA (2014). A multiple imputation strategy for sequential multiple assignment randomized trials. Stat Med.

[87] Schafer JL: Imputation of missing covariates under a multivariate linear mixed model. In: Tech; 1997.

[88] NeCamp T, Kilbourne A, Almirall D: Comparing cluster-level dynamic treatment regimens using sequential, multiple assignment, randomized trials: regression estimation and sample size considerations. arXiv preprint arXiv:160704039 2016. 10.1177/0962280217708654 PMC5802435 28627310

