# Supplementary material for: Adaptive School-based Implementation of CBT (ASIC): clustered-SMART for building an optimized adaptive implementation intervention to improve uptake of mental health interventions in schools
Source: Implement Sci. 2018 Sep 5;13:119. doi: 10.1186/s13012-018-0808-8 (PMC6126013; doi:10.1186/s13012-018-0808-8)
Supplement: Supplementary file 1 — Appendix 1 Determining need for facilitation (randomization criteria). (DOCX 13 kb) [file 13012_2018_808_MOESM1_ESM.docx]

**Appendix 1: Determining Need for Facilitation (Randomization Criteria)**

A key component of the REP strategy is the monitoring of schools and their SPs for implementation success. The TRAILS package includes a monitoring tool for SPs to track and report the number of students identified as needing CBT, the number of students who receive CBT, CBT components delivered by SPs, and barriers to implementation experienced by SPs. The REP TA will encourage SPs to collect this information regularly and may ask SPs to report on these metrics to provide feedback about progress in implementing CBT at their school in relation to other schools (e.g., number of students that have received any CBT, number that have received 3 more components of CBT), as well as to inform content of Technical Assistance calls. All SPs will also receive quarterly reports updating them on their own progress.

In addition, this monitoring component is used to determine whether a school could potentially benefit from Facilitation in Phase 2. Specifically, this determination is based on information gathered via the implementation monitoring process described above as well as a short uptake survey administered to SPs via email (as part of REP monitoring) just prior to the end of Phase 1. This survey includes three components: (1) whether the SP has delivered 4 or more CBT components to any students, either in groups or individual sessions, over the past 8 weeks; and, if yes, (2) the number of students that have received 4 or more CBT components over the past 8 weeks; and (3) which of a list of 10 barriers they have experienced in delivering CBT to students (See Table below).

A school is identified as potentially benefitting from Facilitation if an SP is not providing at least 3 CBT components to at least 10 students (in either individual or group format) OR if SPs at a school report, on average, more than 2 barriers to CBT. These cutoffs are based on our previous studies in which providers who have not offered multiple components of CBT to at least 10 students over 8 weeks are unlikely to offer an adequate dose of CBT over the course of the semester, thus prompting potential use of additional implementation support. Assessment of barriers to sustained implementation as a definition of Facilitation eligibility also ensures that sites that may be currently delivering CBT but face organizational or institutional barriers to sustained delivery can receive it, especially considering potential drift from the EBP after initial deployment of CBT post-training for SPs faced with multiple barriers to implementation. Sites where all SPs are on-target for delivering CBT and report very few organizational barriers to CBT, however, are unlikely to need further implementation support and thus are not considered for Facilitation. Schools where one or more SPs fail to complete the uptake survey or organizational barriers assessment are considered non-adherent and thus also considered as potentially benefiting from Facilitation.

**Table: School Professional Barriers Assessment**

| **In your school, do you believe any of the following are barriers to your delivering CBT to your students? Please select all that apply. If none of these are applicable, please select “None of these are applicable.”** | **Select if applicable** |
| --- | --- |
| 1. Previous CBT delivery experience associated with negative outcomes |  |
| 2. Lack of confidence in identifying students that might benefit from CBT |  |
| 3. Lack of acceptance amongst other school professionals or colleagues |  |
| 4. Lack of support from school administrators |  |
| 5. Lack of students that might benefit from CBT |  |
| 6. Lack of confidence necessary to deliver CBT effectively |  |
| 7. Too many other responsibilities or demands on my time |  |
| 8. Lack of physical resources (e.g., a room for group CBT delivery) |  |
| 9. I don’t believe CBT is effective for improving student mental health |  |
| 10. My efforts to engage students in CBT have been ineffective |  |
| **None of these are applicable** |  |
