# Supplementary material for: Adaptive School-based Implementation of CBT (ASIC): clustered-SMART for building an optimized adaptive implementation intervention to improve uptake of mental health interventions in schools
Source: Implement Sci. 2018 Sep 5;13:119. doi: 10.1186/s13012-018-0808-8 (PMC6126013; doi:10.1186/s13012-018-0808-8)
Supplement: Supplementary file 2 — Appendix 2 School Professional Outcomes Tool. (DOCX 20 kb) [file 13012_2018_808_MOESM2_ESM.docx]

**Appendix 2: School Professional Outcomes Tool**

The School Professional Electronic Student Outcome Survey Tool was created to enable SPs to track information on students’ receipt of CBT sessions in a secure fashion and to ascertain student outcomes.

SPs will be asked to finalize their list of 10 students within 2 weeks after their initial training, and will enter the initial 10 students they identified into this secure, web-based registry in order to ascertain their receipt of CBT. Then each week, SPs, after providing electronic informed consent, will receive an email or text message reminder to fill out a web-based survey to record the total number of CBT sessions delivered that week for each of the identified students in order to populate the registry. The web-based survey will also enable SPs to provide details on whether group or individual sessions were delivered. For group sessions, they will enter how many students were in attendance and whether other SPs participated in the session. For each group or individual session, SPs will also enter in the length of sessions (>15 minutes or <15 minutes), and CBT components covered during the week. We chose a 15 minute cutpoint for sessions in order to capture individual contacts and because they represent the typical contact time for a CBT component to be delivered in schools. Names of students receiving CBT will not be reported. The web-based survey also manages completion of other secondary student outcomes (see section on student outcomes and measures below) and provision of study incentive compensation. This mini-registry and management of student assessments will be embedded in the same web-based tool in order to streamline all field data collection and reduce burden on SPs. SPs will be encouraged to continue to add students to the registry beyond the initial 10 as they identify them as appropriate for CBT treatment over the course of the study.
