# Supplementary material for: Adaptive School-based Implementation of CBT (ASIC): clustered-SMART for building an optimized adaptive implementation intervention to improve uptake of mental health interventions in schools
Source: Implement Sci. 2018 Sep 5;13:119. doi: 10.1186/s13012-018-0808-8 (PMC6126013; doi:10.1186/s13012-018-0808-8)
Supplement: Supplementary file 3 — Appendix 3 Analysis plan. (DOCX 12 kb) [file 13012_2018_808_MOESM3_ESM.docx]

**Appendix 3: Analysis Plan**

Primary Aim

This study design includes four distinct implementation interventions (see Table). The Primary Aim analysis will determine the effect of the most intensive adaptive implementation intervention, #4 which (i) provides all SPs within schools with REP+Coaching in Phase 1 and (ii) augments with Facilitation in Phase 2 for schools that could benefit, versus implementation intervention #1, which provides REP alone to all schools in both phases of intervention (‘control’) on frequency of SP CBT delivery (primary outcome variable). The primary aim analysis is a mean comparison of the total number of CBT sessions delivered by SPs over the course of 18 months between schools in experimental conditions D+F versus schools in experimental conditions A+B in Figure 1. An outline of the possible comparisons is available in the Table below. A weighted comparison between experimental conditions D+F vs. A+B for Primary Aim 1 is required since, as part of the design, a school will contribute differentially to one or more of the 4 embedded implementation interventions in the Table below depending on whether Facilitation is deemed potentially beneficial for the school after Phase 1. To facilitate this weighted comparison, an easy-to-use, marginal, weighted least squares regression approach developed by study investigators will be used [1-4]. The regression analysis model—$\beta_{0} {+\beta}_{1}A_{1}{+\beta}_{2}A_{2}{+\beta}_{3}A_{1}A_{2}+\eta^{T}X$—includes an intercept, a contrast coded (+1/-1) indicator for Phase 1 intervention A1, a contrast-coded indicator for Phase 2 intervention A2, and the interaction between Phase 1 and Phase 2 interventions. To improve statistical efficiency, the regression will adjust for the following baseline school-level measures: school size (>500 students or ≤500 students), percent of students eligible for free/reduced lunch (≥%50 or <50%), location (urban or rural), SP level of education, and job tenure. A robust (sandwich) variance-covariance estimator will be used for the standard errors; this estimator accounts for clustering of SPs within schools and for sampling variability in the proportion of optimally responding schools. The same strategy will be used to analyze the secondary and exploratory outcomes, including change in student mental health outcomes.

The primary contrast for the primary aim is given by ${2(\beta}_{1} {+\beta}_{2})$; specifically, the primary aim will test the null hypothesis that ${2(\beta}_{1} {+\beta}_{2})$ is equal to zero. We will also report the estimates (with 95% confidence intervals) of all parameters in the model, the mean outcome under each of the 4 embedded implementation interventions, and all pair-wise comparisons between the 4 embedded interventions.

Exploratory Aims:

For Exploratory Aim 1 analyses, costs will be estimated for the different embedded implementation interventions. Incremental cost effectiveness ratios (ICERs) will be calculated for each relevant comparison of implementation interventions by dividing the incremental average costs by the incremental average outcomes. The outcomes will include the primary outcome (number of CBT sessions delivered) as well as number of depression or anxiety-free days based on PHQ-9T or GAD-7 score changes between each time point. Confidence intervals and cost-effectiveness acceptability curves will be calculated using standard Monte Carlo methods for simulation/bootstrapping. Analyses will include economic costs associated with student academic outcomes, including absences, suspensions, and grades.

Exploratory Aim 2 analyses will assess whether the implementation intervention effectiveness is moderated by baseline or time-varying SP or school-level factors, to capitalize on school-level heterogeneity to inform the adaptive implementation intervention. From prior literature, we have identified several candidate moderators for testing moderation of the effects of both REP versus REP+Coaching (Phase 1) and Facilitation vs. no Facilitation (Phase 2). We will assess whether (a) the effect of augmenting REP with Coaching is moderated by school-aggregated SP training or baseline perceptions of CBT, school size or percent free/reduced lunch eligible; and (b) among schools that show a potential need for further support, whether the effect of augmentation with Facilitation is moderated by school-level CBT delivery during first 8 weeks post- first randomization, number of barriers to CBT reported 8 weeks post-randomization, satisfaction with current implementation support, or school administrator (leadership) support for adoption of innovation.

Specifically, for the comparison between REP and REP+Coaching, we will examine school-aggregated SP prior training and baseline perceptions of CBT, as well as school size and percent free/reduced lunch-eligible. For Phase 2, amongst schools that could benefit from Facilitation, we will examine whether the effect of augmenting with Facilitation is moderated by school-aggregated CBT delivery during first 8 weeks post-randomization, SP satisfaction with Phase 1 implementation support, number of barriers to CBT reported, or school administrator support for adoption of innovation. A major advantage of the SMART design is the ability to compare baseline *and* time-varying moderators of the effect of Phase 2 implementation strategies among schools that might benefit from Facilitation; this allows us to include variables such as number of CBT sessions delivered during Phase 1 in our moderators analysis for Phase 2. Results of these analyses will be used to construct a more deeply tailored adaptive implementation intervention that further improves uptake, and particularly SP delivery of CBT. To do this, Q-learning [5-8] a generalization of moderated regression analysis to multiple phases of treatment, will be used to build a high-quality, optimal adaptive intervention. Q-learning regression uses a backward induction (dynamic programming) logic that incorporates effects of future treatment decisions in evaluation of tailoring variables (i.e., time-varying moderators) [9] build optimal present treatment decisions. Primary and secondary outcomes, aggregated to the level of the school, will be analyzed with Q-learning [10].

Exploratory Aim 3 analyses will test mechanisms through which the Coaching and Facilitation implementation strategies increase frequency of CBT delivery and improve student mental health outcomes. Coaching is hypothesized to improve CBT fidelity and uptake by increasing SP CBT knowledge, perception and skill; Facilitation is hypothesized to improve CBT uptake by improving SP CBT perception and school administrator support. First, we will examine the effect of Coaching (Phase 1 and 2) and Facilitation (Phase 2) using methods similar to those described in the Primary Aim analysis section. Second, using mediator-analysis methodologies [11-14] we will examine how (a) changes in SP CBT knowledge, perception and skill strategies due to Coaching act as mechanisms by which CBT delivery by SPs increases; and (b) changes in SP CBT perception and school administrator support due to Facilitation acts as mechanisms by which CBT delivery by SPs increases, adjusting for baseline and time-varying factors jointly associated with hypothesized mediators and SP delivery of CBT to students (e.g., change in administrator support for evidence-based practices) via inverse-probability-of-treatment weighting [15-16].

**Table: Adaptive Implementation Interventions Embedded in Study Design**

| **(A1, A2)** | **Implementation Intervention** | **Phase 1 Intervention** | ***Potential Need for Facilitation After Phase 1*** | **Phase 2 Intervention** | **Experimental Conditions**  **(Figure 1)** |
| --- | --- | --- | --- | --- | --- |
| **(+1, -1)** | **#1:** REP only | REP | *Ineligible* | REP | **A+B** |
|  |  |  | *Eligible* | REP |  |
| **(+1, +1)** | **#2:** REP with Facilitation | REP | *Ineligible* | REP | **A+C** |
|  |  |  | *Eligible* | REP + Facilitation |  |
| **(-1, -1)** | **#3:** REP with Coaching | REP + Coaching | *Ineligible* | REP + Coaching | **D+E** |
|  |  |  | *Eligible* | REP + Coaching |  |
| **(-1, +1)** | **#4:** REP with Coaching and Facilitation | REP + Coaching | *Ineligible* | REP + Coaching | **D+F** |
|  |  |  | *Eligible* | REP + Coaching + Facilitation |  |

**Note:** Schools are considered eligible for facilitation after Phase 1 if (1) one or more SPs at the school reports delivering <3 CBT components to <10 students; or (2) on average, SPs report >2 organizational barriers to CBT.

**References:**

1. Orellana L, Rotnitzky A, Robins JM: Dynamic regime marginal structural mean models for estimation of optimal dynamic treatment regimes, Part I: Main content. The international journal of biostatistics 2010, 6(2):1557-4679.
2. Robins J, Orellana L, Rotnitzky A: Estimation and extrapolation of optimal treatment and testing strategies. Statistics in medicine 2008, 27:4678-4721.
3. Nahum-Shani I, Qian M, Almirall D, Pelham WE, Gnagy B, Fabiano GA, Waxmonsky JG, Yu J, Murphy SA: Experimental design and primary data analysis methods for comparing adaptive interventions. Psychological methods 2012, 17:457.
4. NeCamp T, Kilbourne A, Almirall D: Comparing cluster-level dynamic treatment regimens using sequential, multiple assignment, randomized trials: Regression estimation and sample size considerations. Statistical methods in medical research 2017, 26(4):1572-1589.
5. Nahum-Shani I, Qian M, Almirall D, Pelham WE, Gnagy B, Fabiano GA, Waxmonsky JG, Yu J, Murphy SA: Q-learning: A data analysis method for constructing adaptive interventions. Psychological methods 2012, 17:478.
6. Zhao Y-Q, Zeng D, Laber EB, Kosorok MR: New statistical learning methods for estimating optimal dynamic treatment regimes. Journal of the American Statistical Association 2015, 110:583-598.
7. Chakraborty B, Moodie EE: Statistical methods for dynamic treatment regimes. Springer; 2013.
8. Schulte PJ, Tsiatis AA, Laber EB, Davidian M: Q-and A-learning methods for estimating optimal dynamic treatment regimes. Statistical science: a review journal of the Institute of Mathematical Statistics 2014, 29:640.
9. Gail M, Simon R: Testing for qualitative interactions between treatment effects and patient subsets. Biometrics 1985:361-372.
10. Ghosh P, Cheung YK, Chakraborty B: Sample size calculations for cluster SMART designs. In Adaptive Treatment Strategies in Practice: Planning Trials and Analyzing Data for Personalized Medicine. Edited by MR Kosorok EM. Philadelphia, PA; Alexandria, VA: American Statistical Associaton and Society and Society for Industrial and Applied Mathematics; 2016: ASA-SIAM Series on Statistics and Applied Probability.
11. Hong G, Nomi T: Weighting methods for assessing policy effects mediated by peer change. Journal of Research on Educational Effectiveness 2012, 5:261-289.
12. VanderWeele T, Vansteelandt S: Conceptual issues concerning mediation, interventions and composition. Statistics and its Interface 2009, 2:457-468.
13. MacKinnon DP: Integrating mediators and moderators in research design. Research on Social Work Practice 2011, 21:675-681.
14. Hong G: Marginal mean weighting through stratification: A generalized method for evaluating multivalued and multiple treatments with nonexperimental data. Psychological methods 2012, 17:44.
15. Coffman DL: Estimating causal effects in mediation analysis using propensity scores. Structural equation modeling: a multidisciplinary journal 2011, 18:357-369.
16. Coffman DL, Zhong W: Assessing mediation using marginal structural models in the presence of confounding and moderation. Psychological Methods 2012, 17:642.
